# Pemafibrate improves liver dysfunction and non-invasive surrogates for liver fibrosis in patients with non-alcoholic fatty liver disease with hypertriglyceridemia: a multicenter study

**DOI:** 10.1007/s12072-022-10453-1

**Published:** 2022-12-30

**Authors:** Asahiro Morishita, Kyoko Oura, Kei Takuma, Mai Nakahara, Tomoko Tadokoro, Koji Fujita, Joji Tani, Tingting Shi, Takashi Himoto, Miwa Tatsuta, Akio Moriya, Tomonori Senoo, Akemi Tsutsui, Takuya Nagano, Koichi Takaguchi, Masafumi Ono, Tsutomu Masaki

**Affiliations:** 1 Department of Gastroenterology and Neurology, Kagawa University, Kita-gun, Kagawa, 761-0793 Japan; 2 Department of Medical Technology, Kagawa Prefectural University of Health Sciences, Takamatsu, Japan; 3 Department of Gastroenterology, KKR Takamatsu Hospital, Takamatsu, Japan; 4 Department of Gastroenterology, Mitoyo General Hospital, Kanonji, Japan; 5 Department of Hepatology, Kagawa Prefectural Central Hospital, Takamatsu, Japan

**Keywords:** NAFLD, Hypertriglyceridemia, Pemafibrate, Fibrotic biomarkers, FAST score

## Abstract

**Background:**

This retrospective, multicenter study evaluated the effect of pemafibrate treatment on liver function and fibrosis by liver function tests (LFTs) and various fibrotic biomarkers including FibroScan in non-alcoholic fatty liver disease (NAFLD) with hypertriglyceridemia.

**Methods:**

A total of 138 NAFLD patients treated with pemafibrate at three hospitals between September 2018 and April 2021 were included. To evaluate the effect of pemafibrate treatment, FibroScan-aspartate aminotransferase (FAST) score, a novel index of steatohepatitis that can be calculated based on the aspartate aminotransferase (AST) value, controlled attenuation parameter (CAP), and liver stiffness measurement (LSM) was used.

**Results:**

Serum TG levels were significantly decreased 4 weeks after pemafibrate treatment (*p* = 0.003). The levels of AST (*p* = 0.038), alanine aminotransferase (ALT) (*p* = 0.003), and gamma-glutamyl transferase (GGT) (*p* = 0.047) also significantly diminished 12 weeks after pemafibrate administration compared to before administration (*p* < 0.05). However, serum HDL-cholesterol (*p* = 0.193), LDL-cholesterol (*p* = 0.967), and eGFR (*p* = 0.909) levels were not significantly altered 12 weeks after pemafibrate administration. In addition, the fibrosis biomarkers’ Type IV collagen (*p* = 0.753) and FIB-4 index (*p* = 0.333) did not significantly differ, while Autotaxin (*p* = 0.006) and the AST-to-platelet ratio index (APRI) (*p* = 0.003) significantly decreased 48 weeks after pemafibrate administration. No significant reductions in LSM (*p* = 0.959) and CAP (*p* = 0.266) were detected using FibroScan 48 weeks after pemafibrate administration. FAST score was significantly improved (*p* = 0.0475).

**Conclusion:**

Pemafibrate improved LFTs, including fibrotic biomarkers and FAST score, due to the hepatic anti-inflammatory effect, suggesting that pemafibrate may prevent disease progression in NAFLD patients with hypertriglyceridemia.

**Supplementary Information:**

The online version contains supplementary material available at 10.1007/s12072-022-10453-1.

## Introduction

Non-alcoholic fatty liver disease (NAFLD) has become a common public health concern in recent years [1]. The global prevalence of NAFLD is approximately 25%, and has recently been increasing in the Asia–Pacific region [2]. NAFLD manifests in a broad spectrum of conditions, ranging from non-alcoholic fatty liver to non-alcoholic steatohepatitis (NASH), cirrhosis, and hepatocellular carcinoma, making it critical to identify its pathogenesis and establish treatment methods [3–5]. To manage NAFLD, various guidelines have been recommended worldwide (European Association for the Study of the et al. 2016; [7, 8]). However, to date, there have been no recommendations on pharmacotherapy for the treatment of NAFLD/NASH.

Peroxisome proliferator-activated receptors (PPARs) include three types (α, δ, and γ) that form a subfamily of the nuclear receptor superfamily [9]. In particular, increased PPARγ expression is observed in patients with NAFLD, with increased triglyceride accumulation and de novo lipid formation in the liver [10]. On the other hand, PPARα is a heterogeneous molecular target that induces peroxisome proliferation [6] and is established as an important lipid regulator [11, 12]. Activated PPARα induces fatty acid uptake, utilization, and catabolism [13] and may improve NAFLD. Therefore, PPARα modulation is increasingly being considered as an important therapeutic molecules for NAFLD [14].

Pemafibrate (K-877; Palmodia^®^ Tablets, Kowa Co., Nagoya, Japan), a selective PPARα modulator, is highly selective for PPARα. This drug was approved for the treatment of hyperlipidemia in July 2017 and launched in Japan in June 2018 [15]. Pemafibrate is characterized by high selectivity and can therefore be used in reduced doses. Ikeda et al. demonstrated that pemafibrate administration during short-time dramatically improves liver function tests (LFTs) for NAFLD patients with hypertriglyceridemia [16]. Although fibrates demonstrated worsening liver and kidney function test values, pemafibrate improved LFTs and did not augment blood creatinine or diminish the estimated glomerular filtration rate (eGFR), significantly. In addition, several reports have recently demonstrated that pemafibrate can recover liver dysfunction in NAFLD [17–21]. However, its efficacy in NAFLD has not yet fully been elucidated.

Therefore, we retrospectively evaluate the efficacy of pemafibrate on LFTs and non-invasive tests in NAFLD patients with hypertriglyceridemia in a multicenter study.

## Materials and methods

### Study design and protocols

The present study is a multicenter, retrospective, observational study enrolling 266 patients administered pemafibrate from 2018 to 2021 at Kagawa University Hospital, Kagawa Prefectural Central Hospital, or Mitoyo General Hospital. All patients selected for this study were diagnosed as a fatty liver using ultrasonography (US). Patients with chronic hepatitis due to other causes such as hepatitis B virus, hepatitis C virus, autoimmune hepatitis, and primary cholangitis were excluded. Hypertriglyceridemia was diagnosed based on an elevated blood concentration of fasting TG (≥ 150 mg/dL) or non-fasting TG (≥ 175 mg/dL). Patients were prescribed pemafibrate (oral, 0.1 mg, twice a day) and visited the outpatient clinic every 4–12 weeks. The patients also received a biochemical examination to investigate the lipid profile, liver function, and renal function every 4–12 weeks. We carried out transient elastography (FibroScan; ECHOSENS, Paris, France) to examine the liver stiffness measurement (LSM) and controlled attenuation parameter (CAP) at pretreatment, at 12 weeks, 24 weeks, and 48 weeks, since, FibroScan-aspartate aminotransferase (FAST) score was reported to be improved after 48-week pemafibrate administration [19]. Patients who self-discontinued pemafibrate for any reason had a history of drinking (ethanol intake > 20 g/day for female and > 30 g/day for male), or had been taking pemafibrate for a short period (< 1 year) were excluded from the study.

**Fig. 1:**
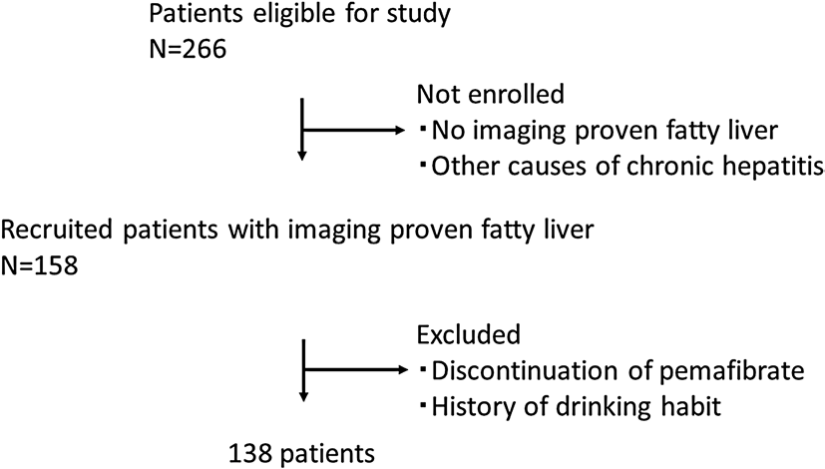
Flowchart of patient selection. Initially, there were 266 eligible patients. 108 patients were not enrolled, because they had not been proven to have fatty liver through imaging. Twenty patients with other causes of chronic hepatitis were also not enrolled. Patients who stopped pemafibrate for any reason and/or with a history of drinking and short duration of using pemafibrate were excluded from the study. Finally, 138 patients were enrolled for this study

According to the European Association for the Study of the Liver (EASL), European Association for the Study of Diabetes (EASD) and European Association for the Study of Obesity (EASO) clinical practice guidelines for the management of non-alcoholic fatty liver disease (European Association for the Study of the et al. 2016), ultimately 138 NAFLD patients (88 male, 50 female) of high-risk fatty liver with metabolic syndrome and increased ALT were selected (Fig. 1). Patients with progressive NASH (bridging fibrosis and cirrhosis) were not confirmed by liver histology in most cases. Patient’s basic characteristics were examined, including sex, age, height, body weight, body mass index (BMI), and information about concomitant medications, which might be effective for NAFLD/NASH: dipeptidyl peptidase-4 (DPP4) inhibitor, metformin, sodium-glucose cotransporter 2 (SGLT2) inhibitor, eicosapentaenoic acid (EPA), statin, ezetimibe, and ursodeoxycholic acid (UDCA). In addition, various fasting laboratory data, such as triglyceride (TG), high-density lipoprotein (HDL) cholesterol, low-density lipoprotein (LDL) cholesterol, aspartate aminotransferase (AST), alanine aminotransferase (ALT), gamma-glutamyl transferase (GGT), HbA1c, fibrosis based on four factors (FIB-4) index [22], Shah et al. [23]; [24], AST-to-platelet ratio index (APRI) [25, 26], eGFR, Type IV collagen, and autotaxin were examined for this study. In addition, LSM, CAP, and FAST score were also evaluated using transient elastography [19]. Patients were prescribed pemafibrate (oral, 0.1 mg, twice a day) and visited the outpatient clinic every 2–8 weeks. The patients also received a biochemical examination to investigate the lipid profile, liver function, and renal function every 1–2 months. The Common Terminology Criteria for Adverse Events version 5.0 was used to evaluate adverse events (AEs) associated with pemafibrate.

### Statistical analysis

GraphPad Prism version 8.4.2 (GraphPad Software, San Diego, USA) was used for statistical analyses. Data are presented as count (%), mean ± standard deviation, or median (25–75th). A comparison between the two treatment groups was performed by the chi-square test. The Student’s *t* test was used to compare numerical data for each group. A value of *p* < 0.05 was considered significant. Univariate analyses for continuous variables were undertaken using the Student’s *t* test, paired *t* test, and Mann–Whitney *U* test.

## Results

### Baseline characteristics of the patients

The median age of the patients was 59 years (range, 21–89 years; Table 1). Thirty-four patients had type 2 diabetes (T2DM) (24.6%). All patients were diagnosed as fatty liver using US. Six patients had been diagnosed with NASH by liver biopsy. Pre-pemafibrate treatment mean laboratory values were as follows: TG 387.3 ± 49.6 mg/dL, HDL-cholesterol 48.6 ± 1.6 mg/dL, LDL-cholesterol 113.7 ± 2.9 mg/dL, AST 42.8 ± 2.3 U/L, ALT 53.8 ± 3.6 U/L, GGT 96.2 ± 9.9 U/L, HbA1c 6.5 ± 0.1%, FIB-4 index 1.95 ± 0.18, APRI 0.72 ± 0.05, eGFR 71.8 ± 21.8, Type IV collagen 5.31 ± 2.6, and autotaxin 55.9 ± 33.5. DPP4 antagonist, metformin, SGLT2 inhibitor, EPA, statin, ezetimibe, and UDCA had been already prescribed in 12 (8.7%), 7 (5.1%), 9 (6.5%), 6 (4.3%), 15 (10.9%), 4 (2.9%), and 12 (8.7%) patients, respectively. Duration of pemafibrate administration was 120 (98–138) in 138 patients treated with pemafibrate (Table1) and 113 (101–128) in 60 patients treated with pemafibrate evaluated by Fibroscan (Table2). Pemafibrate was administered to all patients at 0.1 mg twice per day.

**Table 1:** Characteristics of the 138 patients treated with pemafibrate

Male/female	88/50	
Age (years)	59 (21–89)	
Body height (m)	163.2 (143–185)	
Pre-treatment body weight (kg)	70 (42.5–109.5)	
Pre-treatment BMI (kg/m^2^)	26.0 (18.3–40.6)	
Comorbidities		
T2DM	34	24.6%
Chronic hepatitis B*	0	0%
CAD	8	5.8%
IBD	0	0%
Other	24	17.4%
Biopsy-proven NASH	6	4.3%
Pre-treatment laboratory values		
TG (mg/dL)	387.3±49.6	
HDL-cholesterol (mg/dL)	48.6±1.6	
LDL-cholesterol (mg/dL)	113.7±2.9	
AST (U/L)	42.8±2.3	
ALT (U/L)	53.8±3.6	
GGT (U/L)	96.2±9.9	
HbA1c (%)	6.5±0.1	
FIB-4 index	1.95±0.18	
APRI	0.72±0.05	
eGFR	71.8±21.8	
Type IV collagen	5.31±2.6	
Autotaxin	55.9±33.5	
Concomitant medications		
DPP4 antagonist	12	8.7%
Metformin	7	5.1%
SGLT2 inhibitor	9	6.5%
EPA	6	4.3%
Statin	15	10.9%
Ezetimibe	4	2.9%
UDCA	12	8.7%
Duration of pemafibrate administration (weeks)	120 (98–138)	

APRI, aspartate aminotransferase to platelet ratio index; AST, aspartate aminotransferase; ALT, alanine aminotransferase; BMI, body mass index; HbA1c, hemoglobin A1c; CAD, coronary artery disease; CT, computed tomography; DPP4, dipeptidyl peptidase-4; EPA, eicosapentaenoic acid; GERD, gastroesophageal reflux disease; GGT, gamma-glutamyl transpeptidase; HDL, high-density lipoprotein; IBD, inflammatory bowel disease; LDL, low-density lipoprotein; MRI, magnetic resonance imaging; SGLT2, sodium-glucose cotransporter 2; T2DM, type 2 diabetes mellitus; TG, triglyceride; UDCA, ursodeoxycholic acid; US‚ ultrasonography

*HBV DNA is controlled under detection by nucleotide analog treatment. aHypertension. Data are expressed as median (range) or mean SEM. Numbers in parentheses refer to the percentage of patients

**Table 2:** Characteristics of the 60 patients treated with pemafibrate evaluated by Fibroscan

Male/female	36/24	
Age (years)	57.1 (24–82)	
Body height (m)	163.2 (143–171.8)	
Pre-treatment body weight (kg)	73.5 (53–93.7)	
Pre-treatment BMI (kg/m^2^)	27.8 (18.3–38.2)	
Comorbidities		
T2DM	23	38.3%
Chronic hepatitis B	0	0%
CAD	4	6.7%
IBD	0	0%
Other	10	16.7%
Biopsy-proven NASH	2	3.3%
Pre-treatment laboratory values		
TG (mg/dL)	272.1±30.7	
HDL-cholesterol (mg/dL)	48.9±1.6	
LDL-cholesterol (mg/dL)	120.5±4.7	
AST (U/L)	52.7±3.9	
ALT (U/L)	74.3±6.1	
GGT (U/L)	98.7±11.4	
HbA1c (%)	6.5±0.2	
FIB-4 index	2.0±0.2	
APRI	0.7±0.07	
eGFR (mL/min/1.73m^2^)	66.1±14.5	
Type IV collagen (ng/mL)	4.64±0.9	
Autotaxin (mg/L)	78.3±11.5	
LSM (kPa)	9.33±71	
CAP (dB/m)	318.5±43.3	
FAST Score	0.45±0.22	
Concomitant medications		
DPP4 antagonist	11	18.3%
Metformin	7	11.7%
SGLT2 inhibitor	9	15%
EPA	5	8.3%
Statin	11	18.3%
Ezetimibe	17	28.3%
UDCA	11	18.3%
Duration of pemafibrate administration (weeks)	113 (101–128)	

APRI, aspartate aminotransferase to platelet ratio index; AST, aspartate aminotransferase; ALT, alanine aminotransferase; BMI, body mass index; HbA1c, hemoglobin A1c; CAD, coronary artery disease; CT, computed tomography; DPP4, dipeptidyl peptidase-4; EPA, eicosapentaenoic acid; GERD, gastroesophageal reflux disease; GGT, gamma-glutamyl transpeptidase; HDL, high-density lipoprotein; IBD, inflammatory bowel disease; LDL, low-density lipoprotein; MRI, magnetic resonance imaging; SGLT2, sodium-glucose cotransporter 2; T2DM, type 2 diabetes mellitus; TG, triglyceride; UDCA, ursodeoxycholic acid; US, ultrasonography

### Changes in LFTs and fibrosis markers

**Fig. 2:**
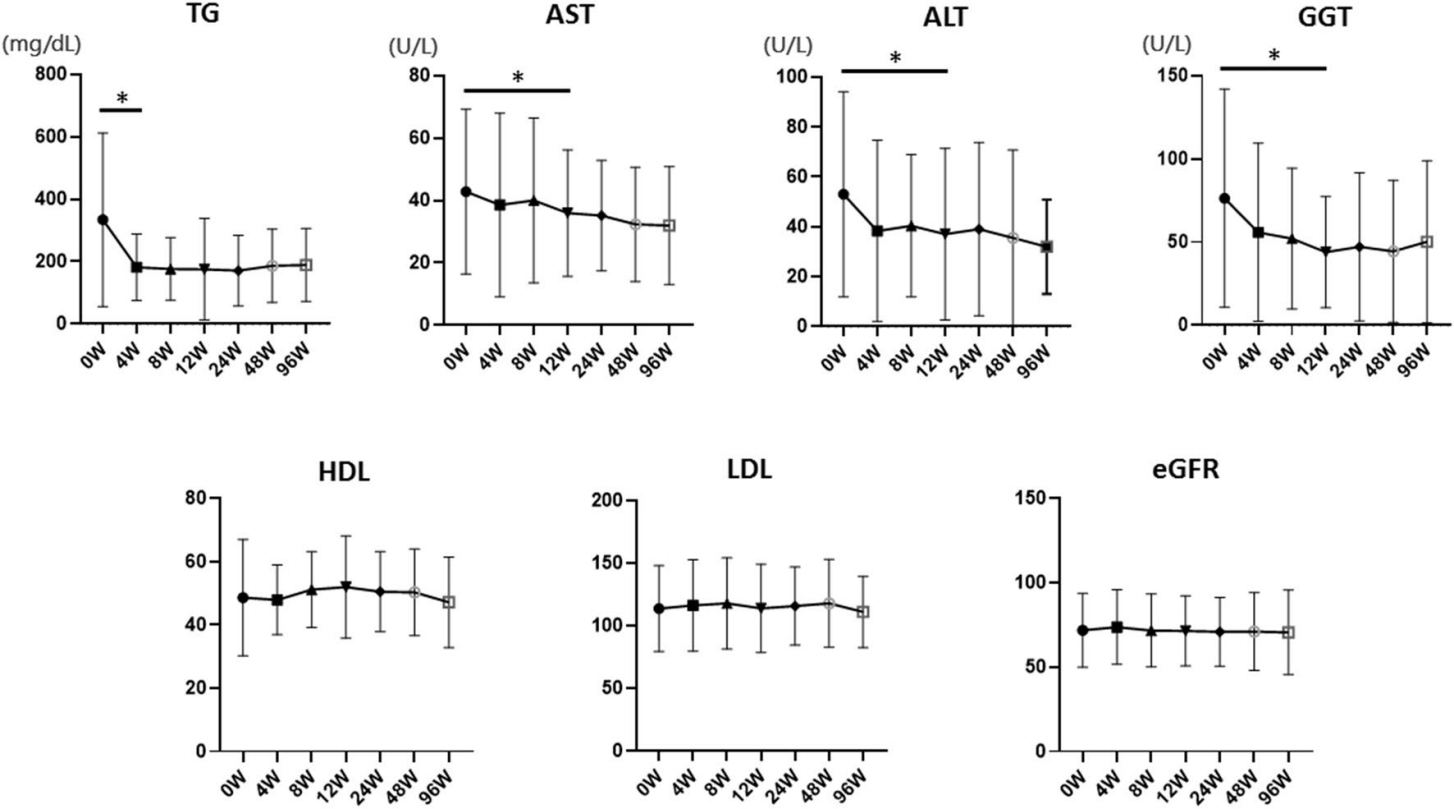
Pre- and post-laboratory data of pemafibrate treatment for 96 weeks. Triglyceride (TG), aspartate aminotransferase (AST), alanine aminotransferase (ALT), and gamma-glutamyl transferase (GGT), high-density lipoprotein cholesterol (HDL), low-density lipoprotein cholesterol (LDL), and estimated glomerular filtration rate (eGFR) were shown. Data are expressed as mean with standard error of the mean (SEM). *p < 0.05

**Fig. 3:**
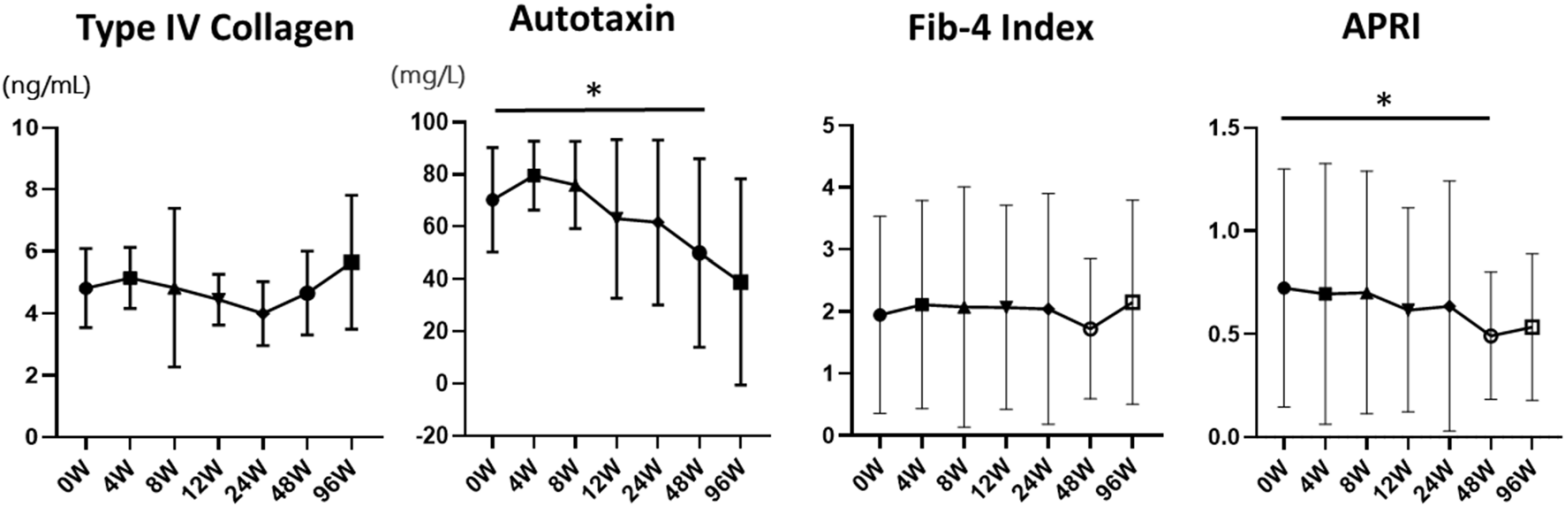
Pre- and post-laboratory data of fibrotic biomarkers on pemafibrate treatment. Type IV collagen, autotaxin, fibrosis based on four factors (FIB-4) index, and aspartate aminotransferase to platelet ratio index (APRI) were shown. Data are expressed as mean with standard error of the mean (SEM). *p < 0.05

Serum TG levels were significantly decreased 4 weeks after pemafibrate treatment (*p* < 0.05) (Fig. 1). The levels of AST (*p* = 0.038), ALT (*p* = 0.003), and GGT (*p* = 0.047) also significantly diminished 12 weeks after pemafibrate administration (Fig. 2). However, serum HDL-cholesterol (*p* = 0.193), LDL-cholesterol (*p* = 0.967), and eGFR (*p* = 0.909) levels were not significantly altered by pemafibrate treatment (Fig. 1). In addition, as biomarkers of liver fibrosis, Type IV collagen (*p* = 0.753) and FIB-4 index (*p* = 0.333) did not significantly differ, while Autotaxin (*p* = 0.006) and APRI (*p* = 0.003) significantly decreased 48 weeks after pemafibrate administration (Fig. 3). BMI and HbA1c were not significantly altered by pemafibrate treatment during the follow-up period (Supplementary Fig. 1).

### Changes in liver steatosis and stiffness

Among the 138 patients, 60 who underwent FibroScan four times within a year were enrolled (Table 2). The median age of these patients was 57 years (range: 24–82 years). Twenty-three patients had type 2 diabetes (T2DM) (38.3%). Two patients were diagnosed with NASH by liver biopsy. The mean laboratory values before pemafibrate administration were as follows: TG 272.1 ± 30.7 mg/dL, HDL-cholesterol 48.9 ± 1.6 mg/dL, LDL-cholesterol 120.5 ± 4.7 mg/dL, AST 52.7 ± 3.9 U/L, ALT 74.3 ± 6.1 U/L, GGT 98.7 ± 11.4 U/L, HbA1c 6.5 ± 0.2%, FIB-4 index 2.0 ± 0.2, APRI 0.7 ± 0.07, eGFR 66.1 ± 14.5 (mL/min/1.73m^2^), Type IV collagen 4.64 ± 0.9 (ng/mL), Autotaxin 78.3 ± 11.5 (mg/L), LSM 9.33 ± 71 (kPa), CAP 318.5 ± 43.3 (dB/m), and FAST score 0.45 ± 0.22. DPP4 antagonist, metformin, SGLT2 inhibitor, EPA, statin, ezetimibe, and UDCA had been already prescribed in 11 (18.3%), 7 (11.7%), 9 (15%), 5 (8.3%), 11 (18.3%), 17 (28.3%), and 11 (18.3%) patients, respectively.

**Fig. 4:**
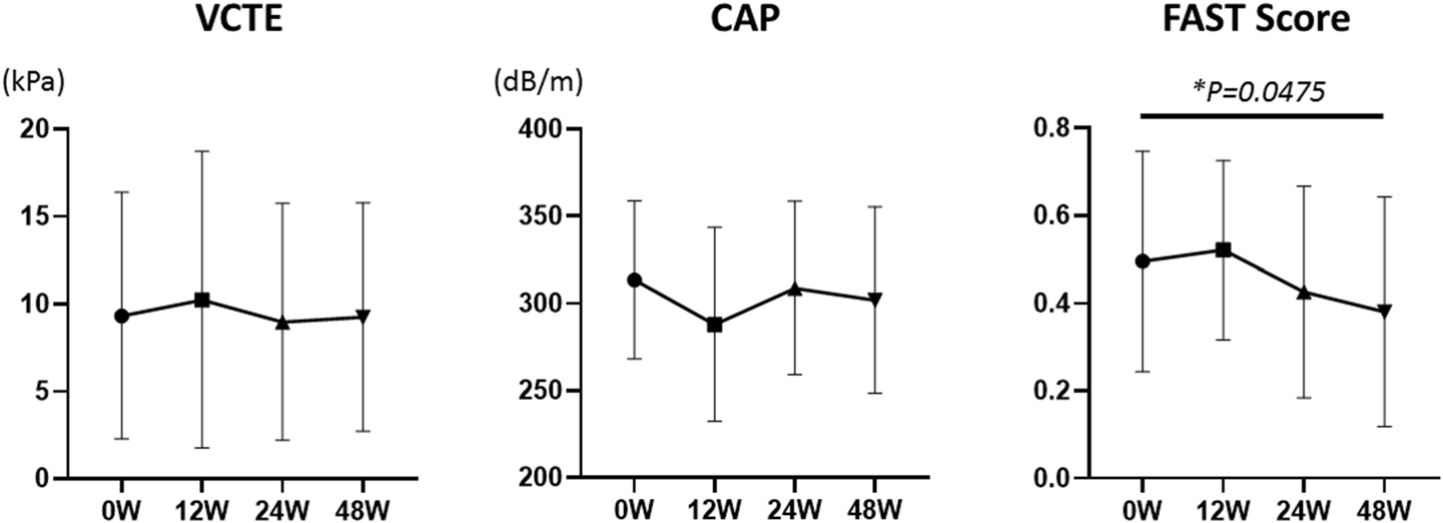
Pre- and post-laboratory data of pemafibrate treatment for 48 weeks. Liver stiffness measurement (LSM) and controlled attenuation parameter (CAP), and FibroScan-aspartate aminotransferase (FAST) score were shown. Data are expressed as mean with standard error of the mean (SEM). *p < 0.05

No significant reductions in LSM and CAP were detected 48 weeks after pemafibrate administration using FibroScan. In contrast, the FAST score was significantly recovered by pemafibrate treatment (*p* = 0.0475; Fig. 4).

## Discussion

In the present study, pemafibrate significantly reduced TG levels in NAFLD with hypertriglyceridemia after 4 weeks of treatment and improved liver dysfunction after 12 weeks of treatment. Surprisingly, 48 weeks of treatment also significantly improved hepatic inflammation and fibrosis markers, as well as hepatic fibrosis in fibroscan. To our knowledge, this is the first multicenter report of a potential effect of pemafibrate on NAFLD patients.

To determine the effect of pemafibrate, we examined if hepatic dysfunction was improved and hepatic fibrosis was prevented by pemafibrate administration in NAFLD patients with hypertriglyceridemia. Previously, several studies have shown the effectiveness of pemafibrate on LFTs, supporting our results [17–21]. Several prospective studies reported that pemafibrate significantly reduced ALT, GGT, and TG levels and increased HDL-cholesterol levels in 20 NAFLD patients in a 12-week single-arm prospective study [21]. They also showed that BMI and insulin resistance were not related to changes in ALT levels. In contrast, a 3-month retrospective observational study of 38 NAFLD patients revealed that pemafibrate significantly decreased ALT, GGT, and TG levels and NAFLD fibrosis score [27] and increased HDL-cholesterol levels [17]. This supports our findings of lower TG levels after 4 weeks and significant reductions in ALT and GGT levels after 12 weeks. Furthermore, in ten biopsy-proven NASH patients treated with pemafibrate, LFTs were significantly improved, especially in NASH patients with high activity and advanced fibrosis [18]. Interestingly, 31 patients with NAFLD treated with pemafibrate and observed for 48 weeks demonstrated improved FAST scores [28] determined by the LSM obtained using vibration-controlled transient elastography (VCTE), estimation of the CAP obtained using a FibroScan device, and estimation of the AST level. The FAST score is expected to reduce unnecessary liver biopsies performed for patients unlikely to have significant disease [19, 28]. In the present study, LSM and CAP did not change significantly, but FAST scores decreased significantly before and 48 weeks after pemafibrate treatment; FAST scores were significantly lower before and 48 weeks after pemafibrate treatment, as measured by the FibroScan test, a comprehensive indicator of liver fibrosis in patients with NASH, which includes (1) liver stiffness, (2) CAP, and (3) AST, and, therefore, may have been the only significant difference. In our present study, FAST score, Autotaxin, and APRI were significantly diminished 48 weeks after pemafibrate administration (Fig. 3, 4). Lee et al. demonstrated that FAST score and APRI were significantly involved in the degree of fibrosis and steatosis [29]. This report supports our data that pemafibrate might improve liver function and fibrosis.

Another recent randomized trial [20] of 118 patients with MRI-proven NAFLD over 72 weeks demonstrated that pemafibrate treatment improved ALT, GGT, and ALP levels as well as cirrhosis. In this randomized controlled trial, there was no baseline statistical difference in liver fat mass; however, fat mass was reduced in the pemafibrate-treated group at 72 weeks. Reports of pemafibrate efficacy against hepatic lipidosis are inconsistent [30, 31]. However, based on the mechanism of pemafibrate, fat mass should be decreased by activating PPARα, and further investigation in humans is warranted. In the present study, Autotaxin and APRI were significantly reduced; APRI included the platelet count. A prospective study by Seko et al. [21] also showed a significant increase in platelet counts, which supports our data. Therefore, platelets might act as critical roles on hemostasis, wound-healing resolution of inflammation, the hepatitis process, and the progression from simple lipemia to NASH [32–34]. Therefore, increased platelet count may be the result of the disappearance of liver inflammation and also explains the significant reduction of APRI.

Autotaxin is highly expressed in the adipose tissue and has been implicated in diet-induced obesity and glucose homeostasis with multiple implications in metabolic disorders. In addition, lysophosphatidic acid (LPA) catalyzed by Autotaxin has been shown to influence stromal and immune cells [35]. Therefore, LPA participates in many processes that are intricately associated with the pathogenesis of different chronic inflammatory diseases. In addition, enhanced expression of Autotaxin has been detected systemically in patients with chronic inflammatory diseases, including chronic liver diseases [35]. Additionally, Autotaxin is degraded by hepatic sinusoidal endothelial cells [36]. Serum Autotaxin levels have been reported to increase in various diseases including certain types of cancer [37–40]. The main mechanism is thought to be the delayed degradation of serum Autotaxin due to the dysfunction of hepatic sinusoidal endothelial cells caused by liver fibrosis [41]. Recently, a correlation between serum Autotaxin concentration and histological severity has been reported in patients with advanced NAFLD [42]. Our data in the present study showed a significant decrease in Autotaxin levels after 48 weeks of pemafibrate treatment, which suggests amelioration of hepatic fibrosis. Further studies using histological analyses may reveal the efficacy of pemafibrate.

Our study was a retrospective, observational study. Selection bias could not be avoided owing to the enrollment method, which registered only patients diagnosed as NAFLD using imaging data. The lack of a control group is another limitation of current study. In addition, the severity of fibrosis which is the recommendation of giving pharmacological treatment to patient with NAFLD (European Association for the Study of the et al. 2016) was not assessed in most cases. This is also the limitation of this study. Liver biopsy is the best diagnostic method for detecting liver fibrosis. However, liver biopsy has several disadvantages including various complications [8]. Non-invasive tools, including scoring systems and various fibrotic biomarkers for assessing fibrosis, have recently been used instead of liver biopsy [43]. The results of the present study are supported by those of previous reports [17–19, 21].

DPP4 antagonists, metformin, and SGLT2 inhibitors, and thiazolidinediones have been demonstrated to have favorable effects on NAFLD in T2DM patients [44, 45]. In the present study, there were 11, 7, and 9 patients who had already taken DPP4 antagonists, metformin, and SGLT2 inhibitors before pemafibrate treatment. No patient had been prescribed thiazolidinediones in this study. No significant differences were detected in the LFTs considering DPP4 antagonists and SGLT2 inhibitors. The patients who had already been treated with DDP4 antagonists and SGLT2 inhibitors before pemafibrate treatment may not have shown differences in LFTs, as their diabetic status had already improved and stabilized. To determine this result, we also compared the pre- and post-HbA1c between with and without DPP4 antagonists or SGLT2 inhibitors. However, no differences were detected between pre-HbA1c (with vs. without DPP4 antagonists or SGLT-2 inhibitors) and post-HbA1c (with vs. without DPP4 antagonists or SGLT2 inhibitors) (data not shown).

As for the drugs for dyslipidemia, several studies have demonstrated the efficacy of β-Hydroxy β-methylglutaryl-CoA reductase inhibitors (statins) on NAFLD/NASH patients [46–49]. Recently, statins are recommended for NAFLD/NASH patients with hypercholesterolemia in the new guidelines of Japan [8]. Statins can ameliorate LFTs; however, consistent histological improvements are still controversial. Pemafibrate is the first fibrate that can be used safely in combination with a statin. In this study, six patients had been prescribed a statin. However, there were no significant differences in the LFTs considering drugs for dyslipidemia, including statins. UDCA is not recommended in the guidelines [8]. Eleven patients had taken UDCA; however, there were no significant differences in the LFTs considering UDCA administration. Furthermore, EPA and ezetimibe were concomitantly used in some participated patients. Previous studies demonstrated that these drugs possibly improve the NAFLD [30–32]. Therefore, their therapeutic efficacy might affect our present results. Accumulating evidences including our study have gradually revealed that pemafibrate can improve liver dysfunction and liver fibrosis in NAFLD patients with hypertriglyceridemia after pemafibrate treatment.

## Conclusions

We demonstrated that pemafibrate improved liver dysfunction assessed by LFTs and liver stiffness evaluated by various fibrotic biomarkers including FibroScan in patients with NAFLD/NASH with hypertriglyceridemia. Therefore, pemafibrate might be a first standard medication for NAFLD.

## Supplementary Information

Below is the link to the electronic supplementary material.

Supplementary file1 Pre and post laboratory data of pemafibrate treatment for 96 weeks. Body Mass Index (BMI) and HbA1c were shown. Data are expressed as mean with standard error of the mean (SEM). *p < 0.05. (TIF 116 KB)

## Data Availability

The data used in the present study are available from the corresponding author upon reasonable request.
