# Supplementary figures and images for: Pemafibrate improves liver dysfunction and non-invasive surrogates for liver fibrosis in patients with non-alcoholic fatty liver disease with hypertriglyceridemia: a multicenter study
Source: Hepatol Int. 2022 Dec 30;17(3):606–14. doi: 10.1007/s12072-022-10453-1 (PMC10224826; doi:10.1007/s12072-022-10453-1)

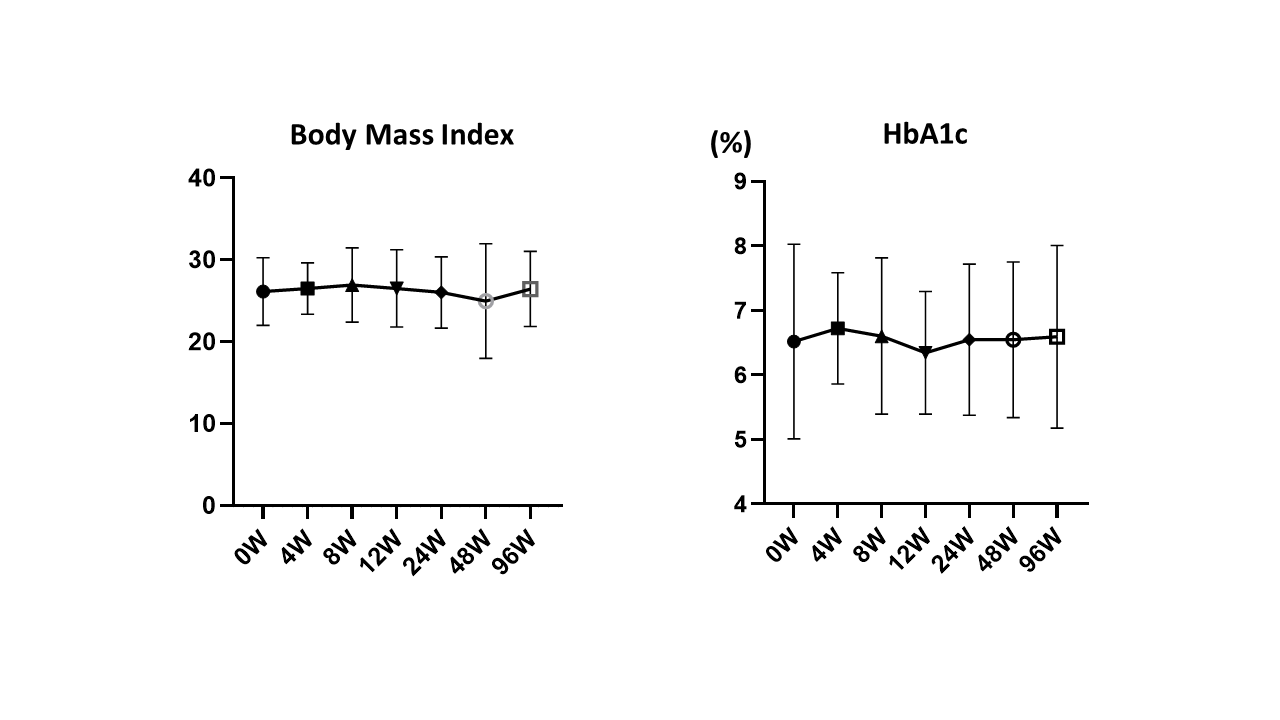

Supplement: Supplementary file 1 — Supplementary file1 Pre and post laboratory data of pemafibrate treatment for 96 weeks. Body Mass Index (BMI) and HbA1c were shown. Data are expressed as mean with standard error of the mean (SEM). *p < 0.05. (TIF 116 KB) [file 12072_2022_10453_MOESM1_ESM.tif]
